# Relation of Vegetarian Dietary Patterns With Major Cardiovascular Outcomes: A Systematic Review and Meta-Analysis of Prospective Cohort Studies

**DOI:** 10.3389/fnut.2019.00080

**Published:** 2019-06-13

**Authors:** Andrea J. Glenn, Effie Viguiliouk, Maxine Seider, Beatrice A. Boucher, Tauseef A. Khan, Sonia Blanco Mejia, David J. A. Jenkins, Hana Kahleová, Dario Rahelić, Jordi Salas-Salvadó, Cyril W. C. Kendall, John L. Sievenpiper

**Affiliations:** ^1^Department of Nutritional Sciences, University of Toronto, Toronto, ON, Canada; ^2^Clinical Nutrition and Risk Factor Modification Center, St. Michael's Hospital, Toronto, ON, Canada; ^3^Toronto 3D Knowledge Synthesis and Clinical Trials Unit, St. Michael's Hospital, Toronto, ON, Canada; ^4^Li Ka Shing Knowledge Institute, St. Michael's Hospital, Toronto, ON, Canada; ^5^Division of Endocrinology and Metabolism, St. Michael's Hospital, Toronto, ON, Canada; ^6^Department of Medicine, Faculty of Medicine, University of Toronto, Toronto, ON, Canada; ^7^Physicians Committee for Responsible Medicine, Washington, DC, United States; ^8^Institute for Clinical and Experimental Medicine, Prague, Czechia; ^9^Vuk Vrhovac University Clinic for Diabetes, Endocrinology and Metabolic Diseases, Merkur University Hospital, Zagreb, Croatia; ^10^School of Medicine, University of Zagreb, Zagreb, Croatia; ^11^CIBER Fisiopatología de la Obesidad y Nutrición (CIBER Obn), Instituto de Salud Carlos III, Madrid, Spain; ^12^Human Nutrition Department, IISPV, Universitat Rovira i Virgili, Reus, Spain; ^13^College of Pharmacy and Nutrition, University of Saskatchewan, Saskatoon, SK, Canada

**Keywords:** vegetarian dietary patterns, vegetarian diets, cardiovascular disease, prospective cohort studies, systematic review, meta-analysis, GRADE

## Abstract

**Background:** Vegetarian dietary patterns are recommended for cardiovascular disease (CVD) prevention and management due to their favorable effects on cardiometabolic risk factors, however, the role of vegetarian dietary patterns in CVD incidence and mortality remains unclear.

**Objective:** To update the European Association for the Study of Diabetes (EASD) clinical practice guidelines for nutrition therapy, we undertook a systematic review and meta-analysis of the association of vegetarian dietary patterns with major cardiovascular outcomes in prospective cohort studies that included individuals with and without diabetes using the Grading of Recommendations, Assessment, Development, and Evaluation (GRADE) approach.

**Methods:** MEDLINE, EMBASE, and Cochrane databases were searched through September 6th, 2018. We included prospective cohort studies ≥1 year of follow-up including individuals with or without diabetes reporting the relation of vegetarian and non-vegetarian dietary patterns with at least one cardiovascular outcome. Two independent reviewers extracted data and assessed study quality (Newcastle-Ottawa Scale). The pre-specified outcomes included CVD incidence and mortality (total CVD, coronary heart disease (CHD) and stroke). Risk ratios for associations were pooled using inverse variance random effects model and expressed as risk ratios (RRs) with 95% confidence intervals (CIs). Heterogeneity was assessed (Cochran Q-statistic) and quantified (*I*^2^-statistic). The overall certainty of the evidence was assessed using GRADE.

**Results:** Seven prospective cohort studies (197,737 participants, 8,430 events) were included. A vegetarian dietary pattern was associated with reduced CHD mortality [RR, 0.78 (CI, 0.69, 0.88)] and incidence [0.72 (0.61, 0.85)] but were not associated with CVD mortality [0.92 (0.84, 1.02)] and stroke mortality [0.92 (0.77, 1.10)]. The overall certainty of the evidence was graded as “very low” for all outcomes, owing to downgrades for indirectness and imprecision.

**Conclusions:** Very low-quality evidence indicates that vegetarian dietary patterns are associated with reductions in CHD mortality and incidence but not with CVD and stroke mortality in individuals with and without diabetes. More research, particularly in different populations, is needed to improve the certainty in our estimates.

**Clinical Trial Registration:** Clinicaltrials.gov, identifier: NCT03610828.

## Introduction

Vegetarian dietary patterns, or vegetarian diets, are defined as diets that exclude meat, poultry, or fish and may or may not include dairy and eggs. Vegetarian dietary patterns are recognized for their health promoting properties as these diets are typically higher in fiber, antioxidants, phytochemicals, and plant protein and lower in saturated fat compared to non-vegetarian dietary patterns (1). Recent systematic reviews and meta-analyses of vegetarian dietary patterns have found that following a vegetarian dietary pattern was associated with reduced risk of coronary heart disease (CHD) in prospective cohort studies, but not with cardiovascular disease (CVD), or stroke (2). Vegetarian dietary patterns also improved cardiometabolic risk factors in randomized controlled trials in individuals with and without diabetes when compared to non-vegetarian dietary patterns (3–5). Currently, the *2015–2020 Dietary Guidelines for Americans* recommend a vegetarian dietary pattern, along with the Mediterranean and healthy U.S. style dietary patterns, as 1 of 3 healthy dietary patterns (6). A number of clinical practice guidelines for diabetes and CVD also recommend vegetarian dietary patterns based on the evidence from systematic reviews and meta-analyses of both prospective cohort studies and randomized controlled trials (2–4). The Canadian Cardiovascular Society (7), Diabetes Canada (8), the American Diabetes Association (9), and Diabetes UK (10) include vegetarian dietary patterns in their clinical practice guidelines for the reduction of cardiovascular disease (CVD) risk factors [including low-density lipoprotein cholesterol (LDL-C), blood pressure (BP) and body weight risk], CVD outcomes, and improvement in glycemic control for individuals with type 2 diabetes.

Despite the evidence supporting the widespread inclusion of vegetarian dietary patterns in dietary guidelines and clinical practice guidelines for nutrition therapy, the European Association for the Study of Diabetes (EASD) (11) has not assessed the evidence for the role of vegetarian dietary patterns in the prevention and management of CVD, an important outcome as CVD, particularly CHD, is the leading cause of premature death in individuals with diabetes (12, 13). To update the clinical practice guidelines for nutrition therapy to include recommendations for the role of vegetarian dietary patterns in the prevention and management of cardiometabolic diseases, the Diabetes and Nutrition Study Group (DNSG) of the EASD commissioned a series of systematic reviews and meta-analyses using the Grading of Recommendations, Assessment, Development, and Evaluation (GRADE) approach, a standard in guideline development to assess the certainty of evidence for important research questions in healthcare. Additional systematic reviews and meta-analyses on vegetarian dietary patterns, diabetes incidence and cardiometabolic risk factors were conducted to update the EASD clinical practice guidelines (5). These systematic reviews and meta-analyses were conducted as the GRADE approach was not previously assessed on this topic. The present systematic review and meta-analysis using GRADE was conducted to address the question of whether the available evidence from prospective cohort studies of vegetarian dietary patterns in comparison with non-vegetarian dietary patterns shows an association with reduced risk of major cardiovascular outcomes in individuals with and without diabetes.

## Methods

### Study Design

We conducted a systematic review and meta-analysis following the methodology from the Cochrane Handbook for Systematic Reviews and Interventions (14). Reporting followed the Meta-analysis of Observational Studies in Epidemiology (MOOSE) (15) and PRISMA guidelines (www.prisma-statement.org). The study protocol was registered on ClinicalTrials.gov (identifier NCT03610828).

### Data Sources and Searches

We searched MEDLINE, EMBASE, and the Cochrane Library (through September 6th, 2018) for relevant prospective cohort studies in humans with no language restrictions. The search strategy is presented in Supplementary Table 1. We supplemented the search with manual searches by identifying cohort studies in the reference lists of included studies.

### Study Selection

We included studies based on the following PICOS (population, intervention, comparator, outcomes, and study design): Population included individuals of all ages with and without diabetes; intervention included vegetarian dietary patterns; comparator included non-vegetarian dietary patterns; outcomes included major cardiovascular outcomes (CVD, CHD, stroke mortality, and incidence) and study design included prospective cohort studies of ≥1-year duration. Vegetarian dietary patterns were considered either as lacto-ovo vegetarian diets, which includes dairy and eggs but excludes all other animal products, or vegan diets, which exclude all animal products including dairy and eggs.

### Data Extraction

Two reviewers (AJG and EV or MS) independently reviewed the articles, extracted relevant data, and assessed risk of bias.

### Risk of Bias

The Newcastle-Ottawa Scale (NOS), a scoring system developed to assess the quality of nonrandomized studies, was used to assess the risk of bias. The studies are judged on three broad perspectives and can receive up to a total of 9 points. The first section is cohort selection (max 4 points), which includes representativeness of the exposed cohorts, selection of the non-exposed cohort and ascertainment of exposure. The second section is the comparability of cohort (max 2 points), which refers to the appropriate inclusion of confounding variables in the analysis. The last section is adequacy of the outcome measures (max 3 points), which includes assessment of outcome and adequacy of follow-up (16). Studies achieving 6 points or more were considered high quality. Differences were reconciled by consensus.

### Outcomes

There were 6 primary outcomes included in the analysis: CVD mortality, CHD mortality, stroke mortality, CVD incidence, CHD incidence, and stroke incidence. CVD incidence and mortality includes all forms of CVD, including both CHD, and stroke outcomes. CHD includes incidence or mortality from atherosclerosis and/or myocardial infarction. Stroke incidence and mortality includes all forms of stroke, including ischemic, hemorrhagic, and unspecified.

### Statistical Analyses

Primary and sensitivity analyses were conducted using Review Manager (RevMan), version 5.3 (Copenhagen, Denmark) and subgroup and publication bias analyses was conducted using STATA software, version 13.0 (College Station, TX, USA). Individual cohort comparison relative risks (RRs) from the most adjusted models were obtained comparing vegetarian vs. non-vegetarian dietary patterns. Hazard Ratios (HRs) were treated as RRs. To obtain summary estimates, we natural log-transformed the RRs and pooled them using DerSimonian-Laird random effects models (17). Heterogeneity was assessed (Cochran Q statistic) and quantified (*I*^2^ statistic). If *I*^2^ was ≥50% and *p* < 0.10, then we interpreted this finding as evidence of substantial heterogeneity (18). Sources of heterogeneity were investigated through sensitivity and subgroup analyses. Sensitivity analyses were performed by systematically removing each study from the meta-analysis with recalculation of the summary estimates in order to assess whether any single study exerted an undue influence on the summary estimates (change of significance and/or direction of association or change of significance of heterogeneity estimate). If ≥10 cohort comparisons were available, a priori subgroup analyses by sex (female, male), type of vegetarian diet (vegan, lacto-ovo), underlying disease status (i.e., diabetes), follow-up (<10 vs. ≥10 years), validation of dietary assessment methods (yes vs. no), NOS (<5 vs. ≥6), and funding source (agency, industry) was conducted using meta-regression. If ≥10 cohort comparisons were available, publication bias by visual inspection of funnel plots and formal testing using the Begg and Egger tests was conducted (19, 20). When publication bias was suspected, adjustment for funnel plot asymmetry was done by imputing missing study data using the Duval and Tweedie trim and fill method (21).

### Grading of the Evidence

The certainty and strength of the evidence was assessed using the Grading of Recommendations, Assessment, Development, and Evaluation (GRADE) system (18, 22–33). Included observational studies started at low-certainty of evidence by default and then were downgraded or upgraded based on pre-specified criteria. Criteria to downgrade certainty included study limitations (weight of studies showing risk of bias by NOS), inconsistency (substantial unexplained inter-study heterogeneity, *I*^2^ ≥ 50% and *P* < 0.10), indirectness (presence of factors relating to the population, exposures and outcomes that limit generalizability), imprecision [95% CIs were wide or crossed a minimally important difference of 5% (RR 0.95–1.05) for all CVD outcomes] and publication bias (significant evidence of small-study effects). Criteria to upgrade included a large effect size (RR > 2 or RR < 0.5 in the absence of plausible confounders), a dose–response gradient and attenuation by plausible confounding effects.

## Results

**Figure 1:**
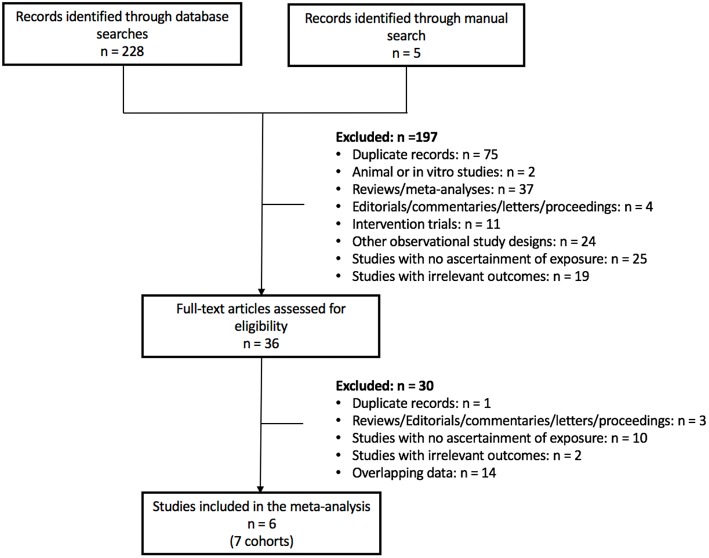
Summary of evidence search and strategy.

**Figure 2:**
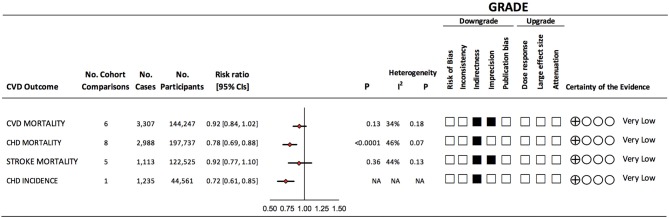
Summary of the pooled effect estimates and certainty of evidence of prospective cohort studies assessing the association between vegetarian dietary patterns and major cardiovascular outcomes. Pooled risk estimate is represented by the diamond. Values of *I*^2^ ≥ 50% indicate substantial heterogeneity (18). Values > 1.0 indicate an adverse association. The Grading of Recommendations, Assessment, Development, and Evaluation (GRADE) of prospective cohort studies are rated as “Low” certainty of evidence and can be downgraded by 5 domains and upgraded by 3 domains. The filled black squares indicate downgrade and/or upgrades for each outcome. CHD, coronary heart disease; CI, confidence interval; CVD, cardiovascular disease; NA, not applicable.

Figure 1 shows the flow of the literature search. Of the 233 reports found, 6 reports with 7 unique prospective cohort studies met eligibility criteria. The 7 unique cohorts include: EPIC-Oxford (34, 35), Heidelberg Study (36), Adventist Health Study-2 (AHS-2) (37), Adventist Mortality Study (AMS) (38), Adventist Health Study-1 (AHS-1) (38), Oxford Vegetarian Study (39), and Health Food Shoppers Study (39) (Table 1). We included sex-specific data from one cohort (37) as separate comparisons. Therefore, we included 6 cohort comparisons for CVD mortality (144,247 participants, 3,307 cases) (34, 36, 37, 39), 8 cohort comparisons for CHD mortality (197,737 participants, 2,988 cases) (34, 36–39), 5 cohort comparisons for stroke mortality (122,525 participants, 1,113 cases) (34, 38, 39), and 1 cohort comparison for CHD incidence (44,561 participants, 1,235 cases) (35) (Figure 2; Table 1). Data from two cohorts (AMS and AHS-1) were extracted from a previous pooled analysis (38). No studies were found that were exclusive to individuals with diabetes or that included subgroup analyses of individuals with diabetes. No studies were found reporting CVD and/or stroke incidence.

**Table 1:** Summary of characteristics of prospective cohort studies assessing the association between vegetarian dietary patterns and major cardiovascular outcomes.

**References**	**Cohort**	**Country**	**No. of participants**	**% vegetarians**	**Outcomes**	**No. of cases**	**Person-years**	**Age range, years**	**Mean follow-up, years (dates)**	**Diet assessment method to determine vegetarian status**	**Exposure**	**Methods of outcome assessment**	**NOS quality score**	**Funding source**
Crowe et al. (35)	EPIC—Oxford	UK	10,602 (M) 33,959 (W)	34	Incident CHD	1,235	517,960	20–89	11.6 (1993–2009)	Validated 130-item FFQ and global questions about meat, fish, dairy, and eggs^f^	Vegetarian/ vegan vs. non-vegetarian	Medical record linkage	7	Agency^a^
Chang- Claude et al. (36)	Heidelberg Study	Germany	858 (M) 1,046 (W)	61.2	CVD + CHD mortality	255 (CVD) 72 (CHD)	NR	10–85	21 (1976–1999)	FFQ included meat, fish, dairy, and eggs	Vegetarian/ vegan vs. non-vegetarian	Death certificates	6	NR
Orlich et al. (37)	AHS-2	USA	25,105 (M) 48, 203 (W)	28.9	CVD + CHD mortality	987 (CVD) 372 (CHD)	NR	57^b^	5.79 (2002–2009)	Validated >200-item FFQ^g^	Vegetarian/ vegan vs. non-vegetarian^d^	National Death Index	6	Agency^a^
Key et al. (38)	Adventist Mortality Study	USA	8,994 (M) 15,544 (W)	41.8	CHD + stroke mortality	598 (CHD) 182 (Stroke)	138, 304	35–80	5.5 (1960–1965)	21-item FFQ	Vegetarian vs. non-vegetarian	Medical record linkage	6	Agency^a^
Key et al. (38)	AHS-1	USA	12, 214 (M) 16, 738 (W)	27.6	CHD + stroke mortality	921 (CHD) 317 (Stroke)	320, 818	25–89	11.1 (1976–1988)	>60-item FFQ	Vegetarian vs. non-vegetarian	Medical record linkage	6	Agency^a^
Key et al. (34)	EPIC—Oxford	UK	11, 324 (M) 35, 930 (W)	34	CVD, CHD + stroke mortality	479 (CVD) 213 (CHD) 159 (Stroke)	506, 620	20–89	8–14^c^ (1993–2007)	Validated 130-item FFQ and global questions about meat, fish, dairy and eggs^f^	Vegetarian/ vegan vs. non-vegetarian^e^	Medical record linkage	7	Agency^a^
Appleby et al. (39)	Oxford Vegetarian Study	UK	4,174 (M) 6,871 (W)	42	CVD, CHD + stroke mortality	469 (CVD) 250 (CHD) 125 (Stroke)	NR	16–89	17.6 (1980–2000)	Never ate meat or fish statement	Vegetarian vs. non-vegetarian	Medical record linkage	5	Agency^a^
Appleby et al. (39)	Health Food Shoppers	UK	4,325 (M) 6,411 (W)	43	CVD, CHD + stroke mortality	1,117 (CVD) 562 (CHD) 330 (Stroke)	NR	16–89	18.7 (1973–1999)	“Are you vegetarian?” question	Vegetarian vs. non-vegetarian	Medical record linkage	6	Agency^a^

*AHS-1, Adventist Health Study-1; AHS-2, Adventist Health Study-2; CHD, coronary heart disease; CVD, cardiovascular disease; EPIC, European Prospective Investigation Into Cancer; FFQ, food frequency questionnaire; M, men; NR, not reported; NOS, Newcastle-Ottawa Scale; USA, United States; UK, United Kingdom; W, women*.

a *Agency funding is that from government, university or not-for-profit health agency sources*.

b *Mean age*.

c *Range of years of follow-up*.

d *Reported disease associations for other forms of vegetarianism in original publication (i.e., vegan, lacto-ovo, pescatarian, semi-vegetarian); only overall vegetarian diet included in current analysis*.

e *Reported disease associations for pescatarians in original publication; combined vegetarian and vegan diet data included in current analysis*.

f *Vegetarian dietary pattern determined by 4 global questions relating to never eating meat, fish, dairy or eggs or by intake of relevant food items reported in FFQ (first 1,300 participants); FFQ validated for nutrients only*.

g *Vegetarian dietary pattern determined by intake of food items of animal origin reported on FFQ; FFQ validated for animal foods/food groups*.

### Study Characteristics

Table 1 shows the characteristics of the included prospective cohort studies. Participants were from the United States, the United Kingdom and Germany, mostly middle-aged (median age range: 33–58 years) and belonged to specific health-conscious groups (i.e., health food store shoppers, members of vegetarian societies, subscribers to vegetarian magazines, and members of the Seventh-Day Adventist church). Prevalence of individuals in the cohorts who followed a vegetarian dietary pattern ranged from 28 to 62%. While no studies excluded individuals with diabetes, only one cohort (EPIC-Oxford) reported prevalence of diabetes (1%) in their cohort (35) and no cohorts conducted subgroup analyses for individuals with diabetes. The mean follow-up durations ranged from 5.5 to 21 years. There were more female than male participants across all outcomes. Ascertainment of cases was done by medical record linkage for all cohorts except one (Heidelberg study), which ascertained mortality through death certificates (36). Vegetarian dietary patterns (combined lacto-ovo and/or vegan) were compared to non-vegetarian, or omnivorous, dietary patterns for all outcomes. This was because only one cohort (AHS-2) reported separate disease associations for different types of vegetarian dietary patterns: lacto-ovo and vegan diets (37). Although dietary intake was assessed by a food frequency questionnaire at baseline in most studies, the assignment of vegetarian status was often based on responses to global questions about the consumption of meat, poultry and/or fish. For example, one cohort (Health Food Shoppers) asked the question “Are you vegetarian?” and one cohort (Oxford Vegetarian Study) asked about never eating meat or fish (39). The EPIC-Oxford cohort assigned vegetarian diet status through four questions on diet groups (meat, fish, dairy, and eggs) or through a food frequency questionnaire (FFQ) (34, 35). All studies were funded by agency alone; except for one cohort (Heidelberg Study), which did not report funding sources (36).

Supplementary Table 2 show the statistical adjustments performed in the included studies. All studies adjusted for the pre-specified primary confounding variable (age). No studies adjusted for all 7 of 9 predefined secondary confounding variables for CVD outcomes (sex, family history of CVD, smoking, markers of overweight/obesity, diabetes, hypertension, dyslipidemia, energy intake, and physical activity) and only one cohort (Oxford Vegetarian Study) adjusted for diabetes status (39).

### Risk of Bias Assessment

Supplementary Table 3 shows the NOS scores for the included prospective cohort studies. Although several studies lost points in several domains, only one cohort (Oxford Vegetarian Study) showed evidence of serious risk of bias (NOS <6) (39).

### Vegetarian Dietary Patterns and CVD Mortality

Figure 2 and Supplementary Figure 1 show the association between vegetarian dietary patterns and CVD mortality (6 cohort comparisons, 144,247 participants and 3,307 cases). We found no significant association (RR 0.92, 95% CI 0.84, 1.02, *p* = 0.13) with no evidence of heterogeneity (*I*^2^ = 34%, *P* = 0.18) when we compared vegetarian dietary patterns to non-vegetarian dietary patterns.

### Vegetarian Dietary Patterns and CHD Mortality

Figure 2 and Supplementary Figure 2 show the association between vegetarian dietary patterns with CHD mortality (8 cohort comparisons, 197 737 participants and 2,988 cases). We found a protective association (RR 0.78, 95% CI 0.69, 0.88, *p* < 0.0001) with no evidence of substantial heterogeneity (*I*^2^ = 46%, *P* = 0.07) when we compared vegetarian dietary patterns to non-vegetarian dietary patterns.

### Vegetarian Dietary Patterns and Stroke Mortality

Figure 2 and Supplementary Figure 3 show the association between vegetarian dietary patterns with stroke mortality (5 cohort comparisons, 122,525 unique participants and 1,113 cases). We found no significant association (RR 0.92, 95% CI 0.77, 1.10, *p* = 0.36) with no evidence of heterogeneity (*I*^2^ = 44%, *P* = 0.13) when we compared vegetarian dietary patterns to non-vegetarian dietary patterns.

### Vegetarian Dietary Patterns and CHD Incidence

Figure 2 shows the association between vegetarian dietary patterns with CHD incidence (1 cohort comparison, 44,561 participants and 1,235 cases). We found a protective association (RR 0.72, 95% CI 0.61, 0.85) in this cohort between vegetarian dietary patterns and CHD incidence. As only one cohort comparison was found, a test for overall effect or heterogeneity was not possible.

### Sensitivity Analyses

Supplementary Table 4 show the sensitivity analyses involving the systematic removal of each study for CVD, CHD and stroke mortality, respectively. For CVD mortality, removing each study did not change the direction or significance of the result, or result in significant heterogeneity. For CHD mortality, the direction or significance of the association did not change, however, individually removing the Adventist Mortality Study, the Heidelberg Study, the Adventist Health Study-2 (men), the Adventist Health Study-2 (women), and EPIC-Oxford introduced significant heterogeneity. Similarly, for stroke mortality, the direction or significance of the association did not change, however, individually removing the Adventist Health Study-1 and the Health Food Shoppers study introduced evidence of substantial heterogeneity.

### Subgroup and Publication Bias Analyses

Subgroup and publication bias analyses were not undertaken as there were <10 cohort comparisons available for each of the CVD outcomes.

### GRADE Assessment

Figure 2 and Supplementary Table 5 show the GRADE assessments for the association between vegetarian dietary patterns and each CVD outcome. The evidence was rated as very low certainty for all outcomes. CVD and stroke mortality were rated as very low certainty owing to downgrades for serious imprecision and indirectness. CHD mortality was rated as very low-certainty owing to downgrades for serious indirectness. All mortality outcomes were downgraded for indirectness as the majority of the studies (comprising of 84–91% of weight in pooled analyses) were done in participants who belonged to specific health-conscious groups (e.g., vegetarian societies, health food store shoppers, subscribers to vegetarian magazines, or were members of the Seventh-day Adventist church). In addition, outcomes were downgraded for indirectness due to the lack of exclusive to or subgroups analyses in patients with diabetes. CHD incidence was rated as very low certainty due to serious indirectness (only one cohort was included in the analysis). Publication bias and a dose-response relationship were not assessed due to limited cohort comparisons.

## Discussion

### Summary of Main Findings

The present systematic review and meta-analysis of prospective cohort studies including individuals with and without diabetes assessed the association between vegetarian dietary patterns (combined lacto-ovo and vegan) and major cardiovascular outcomes. A total of 8 cohort comparisons were included in the analysis: 6 for CVD mortality (144,247 participants and 3,307 cases), 8 for CHD mortality (197,737 participants and 2,988 cases), 5 for stroke mortality (122,525 participants and 1,113 cases), and 1 for CHD incidence (44,561 participants and 1,235 cases) (34–39). Pooled analyses showed that vegetarian dietary patterns were associated with a 22% decrease in CHD mortality and 28% decrease in CHD incidence. Vegetarian dietary patterns were not associated with reductions in CVD and stroke mortality. These findings will provide important evidence to update the clinical practice guidelines for nutrition therapy of the EASD on vegetarian dietary patterns.

### Results in Relation to Other Studies

Our results are consistent with systematic-reviews and meta-analyses of prospective cohorts previously conducted in this area, where vegetarian dietary patterns were associated with a 25% reduced risk of CHD mortality (2). The same study also found that vegetarian dietary patterns were not significantly associated with CVD and stroke mortality. While similar studies were included in both this study and the most recent past systematic reviews and meta-analyses (2), there were slight differences in methodology (i.e., we included sex-specific analysis as different comparisons when possible), and the study did not include a GRADE assessment of the certainty of the evidence, an important assessment for providing evidence-based recommendations for healthcare professionals and to identify knowledge gaps. Systematic reviews and meta-analyses of prospective cohort studies have also consistently shown that increased meat consumption has been associated with increased CVD outcomes, including CHD and stroke (40–43). Furthermore, the evidence from systematic reviews and meta-analyses of randomized controlled trials of intermediate CVD risk factors, including LDL-C, total cholesterol, BP and body weight, found that vegetarian dietary patterns and replacing animal protein with plant protein can significantly reduce these CVD risk factors compared to non-vegetarian dietary patterns or consuming animal protein (3–5, 44, 45).

We were unable to assess the quality of the vegetarian dietary patterns consumed in our pooled analysis due to the limited information on the exposure provided in the cohorts. This highlights an area to be considered in future studies as these aspects may be important for CVD prevention. Recently, an analysis of the Nurse's Health Study and Health Professionals Follow-up Study found that a healthy plant-based dietary pattern (which may or may not be vegetarian) was associated with reduced risk of CHD, whereas an unhealthy plant-based diet high in refined carbohydrates was associated with an increased risk of CHD (46). This finding suggests that the quality of a vegetarian dietary pattern may also be important and that healthy plant-based dietary patterns that include small amounts of meat, poultry or fish may also reduce risk of CHD. We were also unable to separate different types of vegetarian dietary patterns (e.g., vegan) in our pooled analysis due to limited sample size of vegans, highlighting another area to be considered in future studies. Vegan dietary patterns may impact health outcomes differently, as evidenced by an analysis of the Seventh-Day Adventist cohorts which showed that vegan dietary patterns may offer additional CVD protection, particularly in males (47).

### Potential Mechanisms

Several potential mechanisms may explain the protective association found with CHD in our systematic review and meta-analysis and for the cardioprotective effects of vegetarian dietary patterns found in randomized controlled trials. Vegetarian dietary patterns are typically higher in whole grains, fruits, vegetables, nuts, pulses and soy compared to non-vegetarian dietary patterns (48). This difference in eating pattern results in a diet that is typically higher in fiber, phytochemicals (e.g., antioxidants and plant sterols), plant protein, plant-based unsaturated fatty acids, and lower in energy density and saturated fat, factors that have been shown to reduce CVD risk factors and impact overall CVD health through both intrinsic and food displacement mechanisms (1, 5, 45, 49–53). A lower intake of saturated fat may explain the association of reduced risk with CHD, as high saturated fat diets have been shown to increase LDL-C (54), and LDL-C is a known causal factor in the development of atherosclerosis and CHD (55). Other novel mechanisms include reduced intake of heme iron from animal products (56–59) and less trimethyl N-oxide (TMAO) production (60), as higher intake and levels have both been associated with increased CVD events (56–60). Similar mechanisms may also play a role in reduced CVD and stroke incidence or mortality, however, we did not find protective associations of vegetarian dietary patterns with CVD or stroke mortality.

### Strengths and Limitations

The strengths of this study are that we identified all available prospective cohort studies through a systematic search strategy, performed quantitative syntheses and assessed the certainty of the evidence using the GRADE approach. The inability to rule out residual confounding is a limitation inherent in all observational research and explains why prospective cohort studies start at a GRADE of low certainty. Potential sources of residual confounding include reverse causality, the reliability of self-report dietary intake (61) and measurement of the exposure to vegetarian and non-vegetarian dietary patterns, measured and unmeasured confounders included in statistical models, and important collinearity effects from related dietary and lifestyle factors. In particular, as diet was only assessed at baseline in each cohort, we cannot determine if individuals changed their diet over time to include meat or fish, which could lead to misclassification of vegetarian diets during follow-up (62). Some self-reported vegetarians may also consume small amounts of meat and fish, which could also result in misclassification of their diet (63). Another important limitation is indirectness of the study populations. We downgraded the certainty of the evidence for all of the mortality outcomes for serious indirectness, as the majority of the studies (comprising of 84–91% of weight in pooled analyses) were conducted in participants who belonged to specific health-conscious groups (e.g., vegetarian societies, health food store shoppers, subscribers to vegetarian magazines, or were members of the Seventh-day Adventist church) and the populations did not provide subgroup analysis of individuals with diabetes, limiting generalizability. We also downgraded the certainty of the evidence for CHD incidence for indirectness as it was limited to one cohort from the UK. Furthermore, there were no available prospective cohort studies that assessed the relationship of vegetarian dietary patterns with CVD and/or stroke incidence. A final limitation was imprecision. We downgraded CVD and stroke mortality for serious imprecision as the 95% CIs were wide and could not rule out clinically important benefit and/or harm.

Weighing the strengths and limitations of the evidence, the certainty of evidence was considered to be very low certainty for each CVD outcome, owing to downgrades of indirectness due to the limitations of the populations included for all outcomes, and downgrades of imprecision for CVD and stroke mortality.

### Implications

Although the evidence has been rated as very low certainty for all CVD outcomes, if we consider the current study results with findings from systematic reviews and meta-analyses of randomized controlled trials of vegetarian dietary patterns and findings from systematic reviews and meta-analyses of meat consumption in prospective cohort studies, we are provided with further and better quality evidence that vegetarian dietary patterns may be beneficial for CVD risk reduction. The evidence for vegetarian dietary patterns in CVD risk reduction is not only important for clinical practice and dietary guidelines to consider for individual health, but also for growing consumer concerns regarding climate change and animal welfare.

All prospective cohorts included in this analysis were conducted in North America or Europe, where following a vegetarian dietary pattern may be more beneficial for health than other areas in the world where small amounts of meat may improve nutritional status. Globally, in North America and Europe, the number of reported vegetarians is increasing but still remains low, with recent surveys in estimating 10% of the population follows a vegetarian or vegan diet (64–67). This highlights that there is room to shift more individuals toward this healthy dietary pattern as one strategy to improve cardiovascular health, including individuals who have diabetes. Interestingly, recent surveys have reported that 33% of Americans plan to buy more plant-based products in the next year (68), and in Europe recent surveys have indicated that 45% of consumers in Italy and France, 57% in Germany and 61% in Spain report that they regularly include meat-free days in their diet (69). Despite this increased interest in plant-based foods, there is some evidence from a small study in Canada that healthcare professionals are hesitant to recommend vegetarian dietary patterns as they are perceived as too difficult to follow (70). Moreover, the same study reported that 89% of patients living with diabetes did not know a vegetarian dietary pattern was an option to manage their disease and help prevent future CVD, however, 66% reported they would be willing to try the diet with the right support (70). Further, evidence supports that vegetarian dietary patterns have been shown to be as acceptable as other therapeutic diets, suggesting their suitability for long-term use (71–73).

The expanding plant-based food market and availability of vegetarian products in grocery stores and restaurants indicates that this dietary pattern may become easier to follow in the future. As mentioned previously, other implications of a vegetarian dietary pattern for ethical and environmental reasons may also be in line with the values and preferences of some individuals (74, 75). Many scientists have called for significant reductions in the consumption of animal products for sustainable planetary health, in which a vegetarian dietary pattern would fit, as recently described in the global scientific targets of the EAT-*Lancet* Commission (76). Given these consumer trends and concerns, healthcare professionals may have more clients interested in this dietary pattern. Therefore, there is an opportunity for healthcare professionals to consider a vegetarian dietary pattern as one dietary strategy, along with other dietary patterns such as the Mediterranean and Dietary Approaches to Stop Hypertension (DASH), to reduce CVD risk in their patients. Clinical practice guidelines, such as the EASD, should consider developing appropriate resources and tools for healthcare professionals to effectively counsel their patients and address barriers of those interested in following a vegetarian or more plant-based diet (77). Lastly, healthcare professionals can work with their clients to develop a vegetarian dietary pattern that is appropriately planned to ensure adequate nutrient intake, and to ensure that a reliable source of vitamin B12 is included in the diet of those following a vegan dietary pattern (65).

## Conclusions

In conclusion, vegetarian dietary patterns were associated with a reduced risk of CHD mortality and incidence but were not associated with reductions in CVD and stroke mortality in predominantly middle-aged participants with and without diabetes. These findings and GRADE assessment provide a very low certainty of evidence for all CVD outcomes, which will be included in the EASD clinical practice guidelines for nutrition therapy. Sources of uncertainty include the observational study design from which one cannot infer causality, indirectness due to the specific groups studied, and imprecision in the pooled estimates for CVD and stroke mortality. Additional research will have an important influence on the certainty of our estimates. In the absence of randomized controlled trials, additional well-conducted prospective cohort studies in other populations assessing the relationship of vegetarian dietary patterns on CVD outcomes are needed. Future studies should also assess if there are differences between different forms of vegetarianism (e.g., vegan) and the nutritional quality of the vegetarian dietary patterns. There is also a need to assess the role of vegetarian dietary patterns in CVD prevention in patients with diabetes, as there were no studies exclusively in individuals with diabetes and no subgroups analyses conducted in those with diabetes. Lastly, and more importantly, there is a need for more high-quality evidence from large randomized trials assessing the effect of vegetarian dietary patterns on hard CVD outcomes in individuals with and without diabetes.

## Author Contributions

AG, HK, DR, JS-S, CK, and JS conceived and designed the study. All authors analyzed and/or interpreted the data. AG wrote the first draft of the manuscript. All authors revised the article critically for important intellectual content, gave final approval of the version to be published and agreed to be accountable for all aspects of the work.

### Conflict of Interest Statement

CK has received grants or research support from the Advanced Food Materials Network, Agriculture, and Agri-Foods Canada (AAFC), Almond Board of California, American Pistachio Growers, Barilla, Calorie Control Council, Canadian Institutes of Health Research (CIHR), Canola Council of Canada, International Nut and Dried Fruit Council, International Tree Nut Council Research and Education Foundation, Loblaw Brands Ltd, Pulse Canada, Saskatchewan Pulse Growers and Unilever. He has received in-kind research support from the Almond Board of California, American Peanut Council, Barilla, California Walnut Commission, Kellogg Canada, Loblaw Companies, Quaker (Pepsico), Primo, Unico, Unilever, WhiteWave Foods. He has received travel support and/or honoraria from the American Peanut Council, American Pistachio Growers, Barilla, California Walnut Commission, Canola Council of Canada, General Mills, International Nut and Dried Fruit Council, International Pasta Organization, Loblaw Brands Ltd, Nutrition Foundation of Italy, Oldways Preservation Trust, Paramount Farms, Peanut Institute, Pulse Canada, Sabra Dipping Co., Saskatchewan Pulse Growers, Sun-Maid, Tate and Lyle, Unilever and White Wave Foods. He has served on the scientific advisory board for the International Tree Nut Council, International Pasta Organization, McCormick Science Institute, Oldways Preservation Trust, Paramount Farms and Pulse Canada. He is a member of the International Carbohydrate Quality Consortium (ICQC), Executive Board Member of the Diabetes and Nutrition Study Group (DNSG) of the European Association for the Study of Diabetes (EASD), is on the Clinical Practice Guidelines Expert Committee for Nutrition Therapy of the EASD and is a Director of the Toronto 3D Knowledge Synthesis and Clinical Trials foundation. JS-S reports serving on the board of and receiving grant support through his institution from the International Nut and Dried Fruit Council, and Eroski Foundation. Reports serving in the Executive Committee of the Instituto Danone Spain. Has received research support from the Instituto de Salud Carlos III, Spain; Ministerio de Educaciandón y Ciencia, Spain; Departament de Salut Pandública de la Generalitat de Catalunya, Catalonia, Spain; European Commission. Has received research support from California Walnut Commission, Sacramento CA, USA; Patrimonio Comunal Olivarero, Spain; La Morella Nuts, Spain; and Borges S.A., Spain. Reports receiving consulting fees or travel expenses from Danone; California Walnut Commission, Eroski Foundation, Instituto Danone - Spain, Nuts for Life, Australian Nut Industry Council, Nestlandé, Abbot Laboratories, and Font Vella Lanjarandón. He is on the Clinical Practice Guidelines Expert Committee of the European Association for the study of Diabetes (EASD) and served in the Scientific Committee of the Spanish Food and Safety Agency, and the Spanish Federation of the Scientific Societies of Food, Nutrition and Dietetics. He is a member of the International Carbohydrate Quality Consortium (ICQC), and Executive Board Member of the Diabetes and Nutrition Study Group (DNSG) of the EASD. DR is the president of Croatian Society for Diabetes and Metabolic Disorders of Croatian Medical Association. He serves as an Executive Committee member of Croatian Endocrine Society, Croatian Society for Obesity and Croatian Society for Endocrine Oncology. He was a board member and secretary of IDF Europe and currently he is the chair of IDF YLD Programme. He has served as an Executive Committee member of Diabetes and Nutrition Study Group of EASD and currently he serves as an Executive Committee member of Diabetes and Cardiovascular Disease Study Group of EASD. He has served as principal investigator or co-investigator in clinical trials of AstraZeneca, Eli Lilly, MSD, Novo Nordisk, Sanofi Aventis, Solvay, and Trophos. He has received honoraria for speaking or advisory board engagements and consulting fees from Abbott, Amgen, AstraZeneca, Bayer, Boehringer Ingelheim, Eli Lilly, Lifescan–Johnson & Johnson, Novartis, Novo Nordisk, MSD, Merck Sharp and Dohme, Pfizer, Pliva, Roche, Salvus, Sanofi Aventis, and Takeda. DJ has received research grants from Saskatchewan Pulse Growers, the Agricultural Bioproducts Innovation Program through the Pulse Research Network, the Advanced Foods and Material Network, Loblaw Companies Ltd., Unilever, Barilla, the Almond Board of California, Agriculture and Agri-food Canada, Pulse Canada, Kellogg's Company, Canada, Quaker Oats, Canada, Procter and Gamble Technical Center Ltd., Bayer Consumer Care, Springfield, NJ, Pepsi/Quaker, International Nut and Dried Fruit (INC), Soy Foods Association of North America, the Coca-Cola Company (investigator initiated, unrestricted grant), Solae, Haine Celestial, the Sanitarium Company, Orafti, the International Tree Nut Council Nutrition Research and Education Foundation, the Peanut Institute, Soy Nutrition Institute (SNI), the Canola and Flax Councils of Canada, the Calorie Control Council (CCC), the CIHR, the Canada Foundation for Innovation and the Ontario Research Fund. He has received in-kind supplies for trials as a research support from the Almond board of California, Walnut Council of California, American Peanut Council, Barilla, Unilever, Unico, Primo, Loblaw Companies, Quaker (Pepsico), Pristine Gourmet, Bunge Limited, Kellogg Canada, WhiteWave Foods. He has been on the speaker's panel, served on the scientific advisory board and/or received travel support and/or honoraria from the Almond Board of California, Canadian Agriculture Policy Institute, Loblaw Companies Ltd, the Griffin Hospital (for the development of the NuVal scoring system, the Coca-Cola Company, EPICURE, Danone, Diet Quality Photo Navigation (DQPN), Better Therapeutics (FareWell), Verywell, True Health Initiative, Institute of Food Technologists (IFT), Soy Nutrition Institute (SNI), Herbalife Nutrition Institute (HNI), Saskatchewan Pulse Growers, Sanitarium Company, Orafti, the Almond Board of California, the American Peanut Council, the International Tree Nut Council Nutrition Research and Education Foundation, the Peanut Institute, Herbalife International, Pacific Health Laboratories, Nutritional Fundamentals for Health (NFH), Barilla, Metagenics, Bayer Consumer Care, Unilever Canada and Netherlands, Solae, Kellogg, Quaker Oats, Procter and Gamble, the Coca-Cola Company, the Griffin Hospital, Abbott Laboratories, the Canola Council of Canada, Dean Foods, the California Strawberry Commission, Haine Celestial, PepsiCo, the Alpro Foundation, Pioneer Hi-Bred International, DuPont Nutrition and Health, Spherix Consulting and WhiteWave Foods, the Advanced Foods and Material Network, the Canola and Flax Councils of Canada, the Nutritional Fundamentals for Health, Agri-Culture and Agri-Food Canada, the Canadian Agri-Food Policy Institute, Pulse Canada, the Saskatchewan Pulse Growers, the Soy Foods Association of North America, the Nutrition Foundation of Italy (NFI), Nutra-Source Diagnostics, the McDougall Program, the Toronto Knowledge Translation Group (St. Michael's Hospital), the Canadian College of Naturopathic Medicine, The Hospital for Sick Children, the Canadian Nutrition Society (CNS), the American Society of Nutrition (ASN), Arizona State University, Paolo Sorbini Foundation and the Institute of Nutrition, Metabolism, and Diabetes. He received an honorarium from the United States Department of Agriculture to present the 2013 W.O. Atwater Memorial Lecture. He received the 2013 Award for Excellence in Research from the International Nut and Dried Fruit Council. He received funding and travel support from the Canadian Society of Endocrinology and Metabolism to produce mini cases for the Canadian Diabetes Association (CDA). He is a member of the International Carbohydrate Quality Consortium (ICQC). His wife, ALJ, is a director and partner of Glycemic Index Laboratories, Inc., and his sister, CB, received funding through a grant from the St. Michael's Hospital Foundation to develop a cookbook for one of his studies. TK has received research support from the Canadian Institutes of Health Research (CIHR) and an unrestricted travel donation from Bee Maid Honey Ltd. He was an invited speaker at a Calorie Control Council annual general meeting for which he received an honorarium. AG and EV serve as scientific advisors for New Era Nutrition. JS has received research support from the Canadian Foundation for Innovation, Ontario Research Fund, Province of Ontario Ministry of Research and Innovation and Science, Canadian Institutes of health Research (CIHR), Diabetes Canada, PSI Foundation, Banting and Best Diabetes Centre (BBDC), American Society for Nutrition (ASN), INC International Nut and Dried Fruit Council Foundation, National Dried Fruit Trade Association, The Tate and Lyle Nutritional Research Fund at the University of Toronto, The Glycemic Control and Cardiovascular Disease in Type 2 Diabetes Fund at the University of Toronto (a fund established by the Alberta Pulse Growers), and the Nutrition Trialists Fund at the University of Toronto (a fund established by an inaugural donation from the Calorie Control Council). He has received in-kind food donations to support a randomized controlled trial from the Almond Board of California, California Walnut Commission, American Peanut Council, Barilla, Unilever, Unico/Primo, Loblaw Companies, Quaker, Kellogg Canada, and WhiteWave Foods. He has received travel support, speaker fees and/or honoraria from Diabetes Canada, Mott's LLP, Dairy Farmers of Canada, FoodMinds LLC, PepsiCo, International Sweeteners Association, Nestlé, Pulse Canada, Canadian Society for Endocrinology and Metabolism (CSEM), GI Foundation, Abbott, Biofortis, ASN, Health Sciences North, INC Nutrition Research & Education Foundation, European Food Safety Authority (EFSA), and Physicians Committee for Responsible Medicine. He has or has had *ad hoc* consulting arrangements with Perkins Coie LLP, Tate & Lyle, and Wirtschaftliche Vereinigung Zucker e.V. He is a member of the European Fruit Juice Association Scientific Expert Panel. He is on the Clinical Practice Guidelines Expert Committees of Diabetes Canada, European Association for the study of Diabetes (EASD), Canadian Cardiovascular Society (CCS), and Obesity Canada. He serves as an unpaid scientific advisor for the Food, Nutrition, and Safety Program (FNSP) and the Technical Committee on Carbohydrates of the International Life Science Institute (ILSI) North America. He is a member of the International Carbohydrate Quality Consortium (ICQC), Executive Board Member of the Diabetes and Nutrition Study Group (DNSG) of the EASD, and Director of the Toronto 3D Knowledge Synthesis and Clinical Trials foundation. His wife is a former employee of Unilever Canada. HK is the Director of Clinical Research of the Physicians Committee for Responsible Medicine, a nonprofit organization conducting research and education in nutrition. AG, DJ, and HK follow a vegetarian or vegan dietary pattern. The remaining authors declare that the research was conducted in the absence of any commercial or financial relationships that could be construed as a potential conflict of interest.
