# Supplementary material for: Relation of Vegetarian Dietary Patterns With Major Cardiovascular Outcomes: A Systematic Review and Meta-Analysis of Prospective Cohort Studies
Source: Front Nutr. 2019 Jun 13;6:80. doi: 10.3389/fnut.2019.00080 (PMC6585466; doi:10.3389/fnut.2019.00080)
Supplement: Supplementary file 1 [file Data_Sheet_1.docx]

Supplementary Material

RELATION OF VEGETARIAN DIETARY PATTERNS WITH MAJOR CARDIOVASCULAR OUTCOMES: A SYSTEMATIC REVIEW AND META-ANALYSIS OF PROSPECTIVE COHORT STUDIES

***Andrea J Glenn, Effie Viguiliouk, Maxine Seider, Beatrice A Boucher, Tauseef A. Khan, Sonia Blanco Mejia, David JA Jenkins, Hana Kahleová, Dario Raheli*ć*, Jordi Salas-Salvadó, Cyril WC Kendall^*^, John L Sievenpiper^*^***

*** Correspondence:** Corresponding Author: [john.sievenpiper@utoronto.ca](mailto:john.sievenpiper@utoronto.ca); [Cyril.kendall@utoronto.ca](mailto:Cyril.kendall@utoronto.ca)

# Supplementary Figures and Tables.......................................................................................................2-10

**1.1 SUPPLEMENTARY FIGURES**

Supplemental Figure 1. Association between vegetarian dietary patterns and CVD mortality...............................2

Supplemental Figure 2. Association of vegetarian dietary patterns with CHD mortality.......................................3

Supplemental Figure 3. Association of vegetarian dietary patterns with stroke mortality......................................4

**1.2 SUPPLEMENTARY TABLES**

Supplemental Table 1. Search strategy....................................................................................................................5

Supplemental Table 2. Analysis of confounding variables among included prospective cohort studies................6

Supplemental Table 3. Newscastle Ottawa Scale for assessing the quality of prospective cohort studies.............7

Supplemental Table 4. Sensitivity analyses: Systematic removal of each study.....................................................8

Supplemental Table 5. GRADE assessment for CVD outcomes.......................................................................9-10

**2. References.................................................................................................................................................11**

## Supplementary Figures


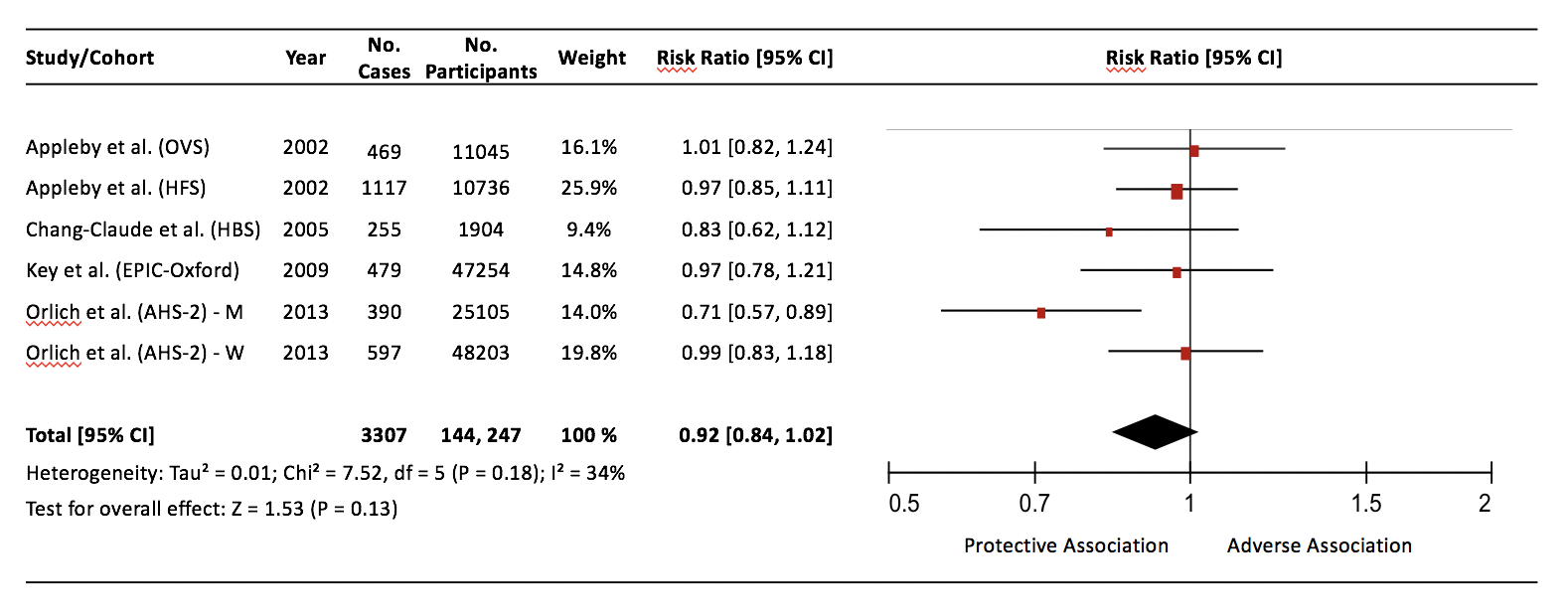


**Supplementary Figure 1.** Association between vegetarian dietary patterns and CVD mortality.

Pooled risk estimate is represented by the diamond. Values of I^2^≥50% indicate substantial heterogeneity (Guyatt et al., 2011e). Values greater than 1.0 indicate an adverse association. AHS-2=Adventist Health Study-2; CVD=cardiovascular disease; CI=confidence interval; EPIC=European Prospective Cohort into Cancer and Nutrition; HBS=Heidelberg Study; HFS=Health Food Shoppers; M=men; OVS=Oxford Vegetarian Study; W=women.


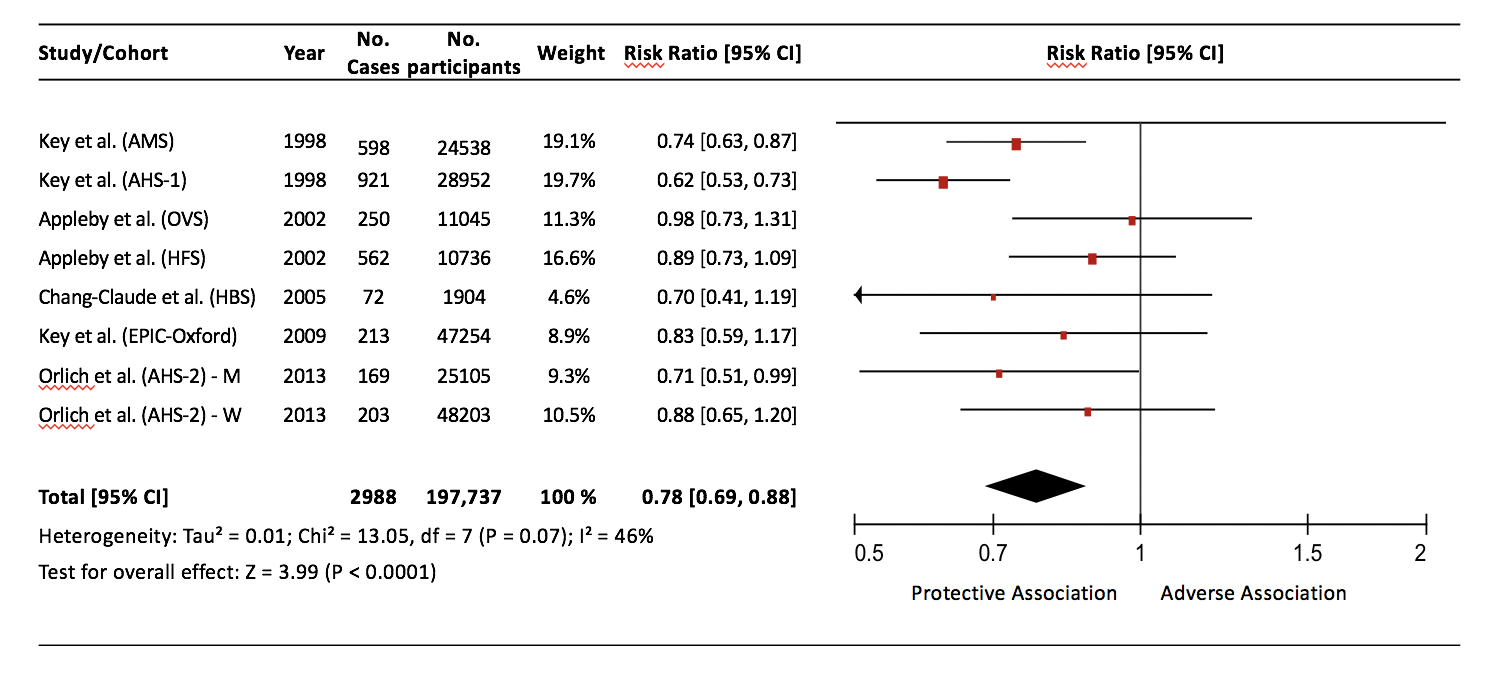


**Supplementary Figure 2.** Association between vegetarian dietary patterns and CHD mortality.

Pooled risk estimate is represented by the diamond. Values of I^2^≥50% indicate substantial heterogeneity (Guyatt et al., 2011e). Values greater than 1.0 indicate an adverse association. AHS-1=Adventist Health Study-1; AHS-2=Adventist Health Study-2; AMS=Adventist Mortality Study; CHD=coronary heart disease; CI=confidence interval; EPIC=European Prospective Cohort into Cancer and Nutrition; HBS=Heidelberg Study; HFS=Health Food Shoppers; M=men; OVS=Oxford Vegetarian Study; W=women.


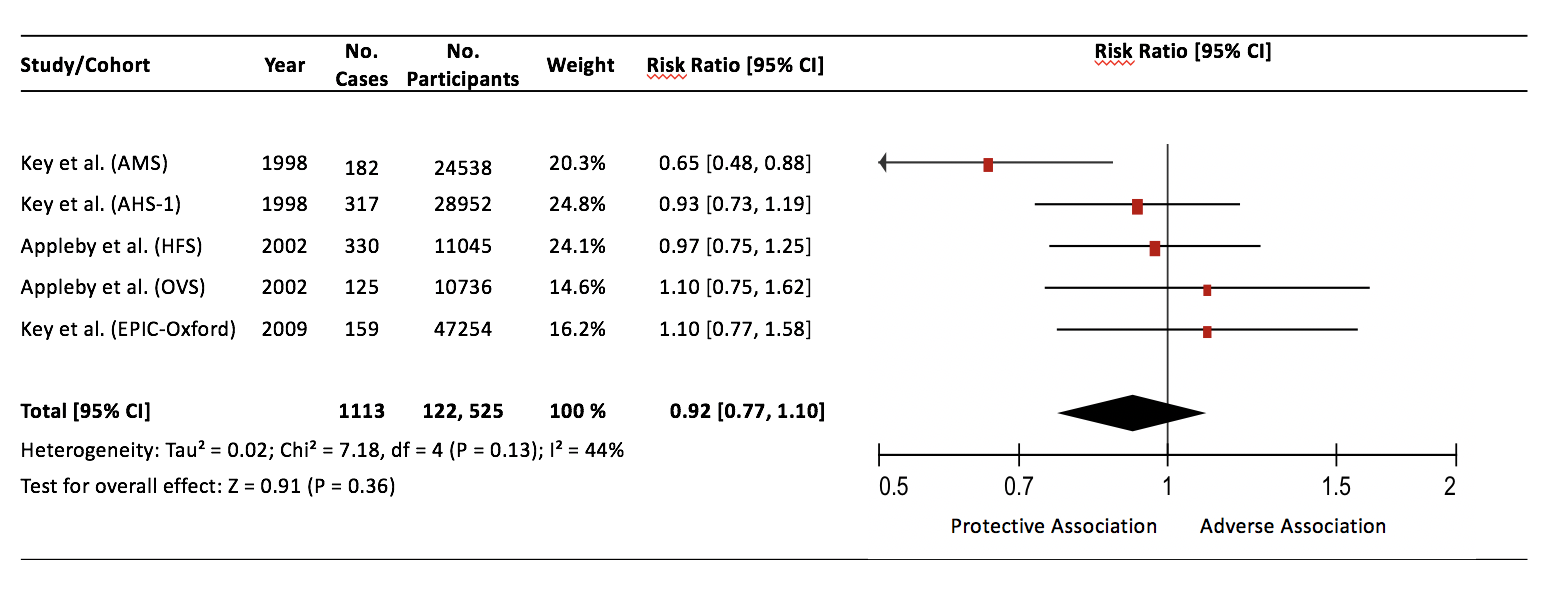


Supplementary Figure 3. Association of vegetarian dietary patterns with stroke mortality

Pooled risk estimate is represented by the diamond. Values of I^2^≥50% indicate substantial heterogeneity (Guyatt et al., 2011e). Values greater than 1.0 indicate an adverse association. AHS-1=Adventist Health Study-1; AMS=Adventist Mortality Study; CI=confidence interval; EPIC=European Prospective Cohort into Cancer and Nutrition; HFS=Health Food Shoppers; OVS=Oxford Vegetarian Study.

**1.2 Supplementary Tables**

| **Medline**: November 1st, 2017.  Updated September 6th, 2018. | | **Embase**: November 1st, 2017.  Updated September 6th, 2018. | | **Cochrane**: November 1st, 2017.  Updated September 6th, 2018. | |
| --- | --- | --- | --- | --- | --- |
| 1 | plant-based diet*.mp | 1 | plant-based diet*.mp | 1 | plant-based diet*.mp |
| 2 | (plant adj1 diet).mp | 2 | (plant adj1 diet).mp | 2 | (plant adj1 diet).mp |
| 3 | exp diet,vegetarian/ | 3 | exp vegetarian diet/ | 3 | exp diet,vegetarian/ |
| 4 | vegetarian*.mp | 4 | exp vegetarian/ | 4 | vegetarian*.mp |
| 5 | vegan*.mp | 5 | vegetarian*.mp | 5 | vegan*.mp |
| 6 | lactoovo*.mp | 6 | vegan*.mp | 6 | lacto-ovo*.mp |
| 7 | lacto-ovo*.mp | 7 | lactoovo*.mp | 7 | or/1-6 |
| 8 | ovolacto*.mp | 8 | lacto-ovo*.mp |  |  |
| 9 | ovo-lacto*.mp | 9 | ovolacto*.mp | 8 | cohort.mp. |
| 10 | or/1-9 | 10 | ovo-lacto*.mp | 9 | exp Prospective Studies/ |
|  |  | 11 | or/1-10 | 10 | (prospective adj2 (cohort or study)).mp. |
| 11 | cohort.mp. |  |  | 11 | exp follow-up studies/ |
| 12 | exp prospective study/ | 12 | cohort.mp. | 12 | exp multivariate analysis/ |
| 13 | (prospective adj2 (cohort or study)).mp. | 13 | exp prospective study/ | 13 | exp proportional hazards models/ |
| 14 | exp follow-up studies/ | 14 | (prospective adj2 (cohort or study)).mp. | 14 | follow up study.mp. |
| 15 | exp multivariate analysis/ | 15 | exp multivariate analysis/ | 15 | (longitudinal adj2 study).mp. |
| 16 | exp proportional hazards models/ | 16 | exp proportional hazards models/ | 16 | or/8-15 |
| 17 | follow up study.mp. | 17 | follow up study.mp. |  |  |
| 18 | (longitudinal adj2 study).mp. | 18 | (longitudinal adj2 study).mp. | 17 | cardiovascular disease.mp. |
| 19 | or/11-18 | 19 | or/12-18 | 18 | cvd.mp. |
|  |  |  |  | 19 | (coronary adj2 disease).mp. |
| 20 | cardiovascular disease.mp. | 20 | cardiovascular disease.mp. | 20 | exp coronary disease/ |
| 21 | cvd.mp. | 21 | cvd.mp. | 21 | cerebrovascular.mp. |
| 22 | (coronary adj2 disease).mp. | 22 | (coronary adj2 disease).mp. | 22 | cerebral vascular.mp. |
| 23 | exp coronary disease/ | 23 | exp coronary disease/ | 23 | exp brain ischemia/ |
| 24 | cerebrovascular.mp. | 24 | cerebrovascular.mp. | 24 | exp stroke/ |
| 25 | cerebral vascular.mp. | 25 | cerebral vascular.mp. | 25 | exp cerebrovascular disorders/ |
| 26 | exp brain ischemia/ | 26 | exp brain ischemia/ | 26 | exp intracranial arterial diseases/ |
| 27 | exp stroke/ | 27 | exp stroke/ | 27 | exp myocardial infarction/ |
| 28 | exp cerebrovascular disorders/ | 28 | exp cerebrovascular disorders/ | 28 | myocardial infarction.mp. |
| 29 | exp intracranial arterial diseases/ | 29 | exp intracranial arterial diseases/ | 29 | exp myocardial ischemia/ |
| 30 | exp myocardial infarction/ | 30 | exp myocardial infarction/ | 30 | myocardial ischemia.mp. |
| 31 | myocardial infarction.mp. | 31 | myocardial infarction.mp. | 31 | or/17-30 |
| 32 | exp myocardial ischemia/ | 32 | exp myocardial ischemia/ |  |  |
| 33 | myocardial ischemia.mp. | 33 | myocardial ischemia.mp. | 32 | and 7, 16, 31 |
| 34 | or/51-64 | 34 | or/20-34 |  |  |
|  |  |  |  |  |  |
| 35 | and 10, 19, 34 | 35 | and 11, 19, 34 |  |  |

**Supplementary Table 1**. Search Strategy

| **Cohort** | **AHS-1 & AMS** | **Health Food Shoppers** | **Oxford Vegetarian Study** | **Heidelberg Study** | **EPIC-Oxford** | **AHS-2** | **EPIC-Oxford** |
| --- | --- | --- | --- | --- | --- | --- | --- |
| **Author** | **Key et al., 1998** | **Appleby et al., 2002** | **Appleby et al., 2002** | **Chang-Claude et al., 2005** | **Key et al., 2009** | **Orlich et al., 2013** | **Crowe et al., 2013** |
| **Pre-specified primary confounding variables** |  |  |  |  |  |  |  |
| Age | x | x | x | x | x | x | x |
| **Pre-specified secondary confounding variables** |  |  |  |  |  |  |  |
| Sex | x | x | x | x | x | x | x |
| Family history of CVD |  |  |  |  |  |  |  |
| Smoking | x | x | x | x | x | x | x |
| Markers of overweight/obesity (BMI, weight, WC, waist to hip ratio) |  |  |  |  |  | x | x |
| Diabetes |  |  | x |  |  |  |  |
| Hypertension |  |  |  |  |  |  |  |
| Dyslipidemia |  |  |  |  |  |  |  |
| Energy intake |  |  |  |  |  | x |  |
| Physical activity |  |  |  | x |  | x | x |
| **Other confounding variables** |  |  |  |  |  |  |  |
| Method of recruitment |  |  |  |  | x |  | x |
| Region |  |  |  |  |  | x | x |
| Education |  |  |  | x |  | x | x |
| SES |  |  |  |  |  |  | x |
| Oral contraceptive use |  |  |  |  |  |  | x |
| HRT use |  |  |  |  |  | x | x |
| Alcohol |  |  |  | x | x | x |  |
| Income |  |  |  |  |  | x |  |
| Marital status |  |  |  |  |  | x |  |
| Sleep |  |  |  |  |  | x |  |
| Menopausal status |  |  |  |  |  | x |  |
| Race/ethnicity |  |  |  |  |  | x |  |
| CVD |  |  | x |  |  |  |  |
| Cancer |  |  | x |  |  |  |  |

Supplementary Table 2. Analysis of confounding variables among included prospective cohort studies. AHS-1=Adventist Health Study-1; AHS-2=Adventist Health Study-2; AMS=Adventist Mortality Study; EPIC=European Prospective Cohort into Cancer and Nutrition.

| **Study** | **Selection (max 4)** | | | | **Outcome (max 3)** | | | **Comparability (max 2)** | | **Total** |
| --- | --- | --- | --- | --- | --- | --- | --- | --- | --- | --- |
|  | **Representativeness of the exposed cohort** | **Selection of the non-exposed cohort** | **Ascertainment of exposure** | **Demonstration that outcome of interest was not present at start of study** | **Assessment of outcome** | **Was follow-up long enough for outcomes to occur** | **Adequacy of follow-up of cohort** | **Study controls for primary confounding variable** | **Study controls for secondary confounding variables** |  |
| Key et al., 1998 (AMS) | 0 | 1 | 0 | 1 | 1 | 1 | 1 | 1 | 0 | 6 |
| Key et al., 1998 (AHS-1) | 0 | 1 | 0 | 1 | 1 | 1 | 1 | 1 | 0 | 6 |
| Appleby et al., 2002 (OVS) | 0 | 0 | 0 | 1 | 1 | 1 | 1 | 1 | 0 | 5 |
| Appleby et al., 2002 (HFS) | 0 | 1 | 0 | 1 | 1 | 1 | 1 | 1 | 0 | 6 |
| Chang-Claude et al., 2005 (HBS) | 0 | 1 | 0 | 1 | 1 | 1 | 1 | 1 | 0 | 6 |
| Key et al., 2009 (EPIC-Oxford) | 1 | 1 | 0 | 1 | 1 | 1 | 1 | 1 | 0 | 7 |
| Crowe et al., 2013  (EPIC-Oxford) | 1 | 1 | 0 | 1 | 1 | 1 | 1 | 1 | 0 | 7 |
| Orlich et al., 2013 (AHS-2) | 0 | 1 | 0 | 1 | 1 | 1 | 1 | 1 | 0 | 6 |

Supplementary Table 3. Newcastle Ottawa Scale (NOS) for assessing the quality of prospective cohort studies

AHS-1=Adventist Health Study-1; AHS-2=Adventist Health Study-2; AMS=Adventist Mortality Study; EPIC=European Prospective Cohort into Cancer and Nutrition; HBS=Heidelberg Study; HFS=Health Food Shoppers; OVS=Oxford Vegetarian Study

| **Removal of** | **RR [95% CI], P-value** | **Heterogeneity** |
| --- | --- | --- |
| **CVD Mortality** | | |
| Appleby et al., 2002 (OVS) | 0.91 [0.81, 1.02], P=0.10 | I² = 42%, P=0.14 |
| Appleby et al., 2002 (HFS) | 0.91 [0.80, 1.03], P=0.14 | I² = 43%, P=0.13 |
| Chang-Claude et al., 2005 (HBS) | 0.93 [0.84, 1.04], P=0.23 | I² = 42%, P=0.14 |
| Key et al., 2009 (EPIC-Oxford) | 0.91 [0.81, 1.03], P=0.14 | I² = 46%, P=0.12 |
| Orlich et al., 2013 (AHS-2: M) | 0.97 [0.89, 1.05], P=0.46 | I² = 0%, P=0.87 |
| Orlich et al., 2013 (AHS-2: W) | 0.91 [0.80, 1.02], P=0.12 | I² = 43%, P=0.14 |
| **CHD Mortality** | | |
| Key et al., 1998 (AMS) | 0.79 [0.68, 0.92], P=0.003 | I² = 54%, P=0.04 |
| Key et al., 1998 (AHS-1) | 0.82 [0.74, 0.90], P=<0.0001 | I² = 0%, P=0.57 |
| Appleby et al., 2002 (OVS) | 0.75 [0.67, 0.85], P=<0.0001 | I² = 38%, P=0.14 |
| Appleby et al., 2002 (HFS) | 0.76 [0.66, 0.86], P=<0.0001 | I² = 40%, P=0.12 |
| Chang-Claude et al., 2005 (HBS) | 0.78 [0.69, 0.89], P=0.0003 | I² = 54%, P=0.04 |
| Key et al., 2009 (EPIC-Oxford) | 0.77 [0.68, 0.89], P=0.0002 | I² = 53%, P=0.05 |
| Orlich et al., 2013 (AHS-2: M) | 0.79 [0.69, 0.90], P=0.0007 | I² = 53%, P=0.04 |
| Orlich et al., 2013 (AHS-2: W) | 0.77 [0.67, 0.88], P=0.0001 | I² = 50%, P=0.06 |
| **Stroke Mortality** | | |
| Key et al., 1998 (AMS) | 0.99 [0.86, 1.15], P=0.93 | I² = 0%, P=0.83 |
| Key et al.,1998 (AHS-1) | 0.92 [0.72, 1.18], P=0.51 | I² = 58%, P=0.07 |
| Appleby et al., 2002 (HFS) | 0.91 [0.72, 1.15], P=0.43 | I² = 56%, P=0.08 |
| Appleby et al., 2002 (OVS) | 0.89 [0.73, 1.09], P=0.27 | I² = 52%, P=0.10 |
| Key et al., 2009 (EPIC-Oxford) | 0.89 [0.73, 1.09], P=0.26 | I² = 50%, P=0.11 |

**Supplementary Table 4**. Sensitivity analyses: Systematic removal of each study.

AHS-1=Adventist Health Study-1; AHS-2=Adventist Health Study-2; AMS=Adventist Mortality Study; CVD=cardiovascular disease; CHD=coronary heart disease; EPIC=European Prospective Cohort into Cancer and Nutrition; HBS=Heidelberg Study; HFS=Health Food Shoppers; M=men; OVS=Oxford Vegetarian Study; RR=relative risk; W=women

| **Certainty assessment** | | | | | | | | **Relative risk (95% CI)** | **Certainty** |
| --- | --- | --- | --- | --- | --- | --- | --- | --- | --- |
| **Outcome** | **No. cohort comparisons** | **Study design** | **Risk of bias** | **Inconsistency** | **Indirectness** | **Imprecision** | **Other**  **considerations** |  |  |
| **CVD mortality** | 6 | observational studies | not serious | not serious | serious^a^ | serious^b^ | none | **RR 0.92** (0.84, 1.02) | ⨁◯◯◯ VERY LOW |
| **CHD mortality** | 8 | observational studies | not serious | not serious | serious^c^ | not serious | none | **RR 0.78** (0.69, 0.88) | ⨁◯◯◯ VERY LOW |
| **Stroke mortality** | 5 | observational studies | not serious | not serious | serious^d^ | serious^e^ | none | **RR 0.92** (0.77, 1.10) | ⨁◯◯◯ VERY LOW |
| **CHD incidence** | 1 | observational studies | not serious | N/A^f^ | serious^g^ | not serious | none | **RR 0.72** (0.61, 0.85) | ⨁◯◯◯ VERY LOW |

Supplementary Table 5. GRADE assessment for CVD outcomes

a. Serious indirectness for CVD mortality since studies comprising >50% weight in the pooled analysis (85.2%) were conducted in participants who were not representative of the general population. [Participants were either (1) part of the Vegetarian Society of the UK and the news media, with non-vegetarians recruited by the vegetarian participants from among their friends and relatives (OVS cohort); (2) customers of health food shops, members of vegetarian societies and readers of relevant magazines (HFS cohort); (3) individuals following a vegetarian or ‘‘healthy’’ lifestyle who were initially recruited from readers of relevant vegetarian magazines (HBS cohort); (4) Seventh-day Adventists (AHS-2 cohort).] In addition, there were no studies exclusive to or that included subgroup analyses in the diabetes population.

b. Serious imprecision for CVD mortality, as the 95% CIs (0.84, 1.02) overlap with the minimally important difference for clinical benefit (RR=0.95).

c. Serious indirectness for CHD mortality since studies comprising >50% weight in the pooled analysis (91.1%) were conducted in participants who were not representative of the general population. [Participants were either (1) part of the Vegetarian Society of the UK and the news media, with non-vegetarians recruited by the vegetarian participants from among their friends and relatives (OVS cohort); (2) customers of health food shops, members of vegetarian societies and readers of relevant magazines (HFS cohort); (3) individuals following a vegetarian or ‘‘healthy’’ lifestyle who were initially recruited from readers of relevant vegetarian magazines (HBS cohort); (4) Seventh-day Adventists (AMS, AHS-1, AHS-2 cohorts).] In addition, there were no studies exclusive to or that included subgroup analyses in the diabetes population.

d. Serious indirectness for stroke mortality since studies comprising >50% weight in the pooled analysis (83.8%) were conducted in participants who were not representative of the general population. [Participants were either (1) part of the Vegetarian Society of the UK and the news media, with non-vegetarians recruited by the vegetarian participants from among their friends and relatives (OVS cohort); (2) customers of health food shops, members of vegetarian societies and readers of relevant magazines (HFS cohort); (3) Seventh-day Adventists (AMS, AHS-1 cohorts).] In addition, there were no studies exclusive to or that included subgroup analyses in the diabetes population.

e. Serious imprecision for stroke mortality, as the 95% CIs (0.77, 1.10) overlap with the minimally important difference for clinical benefit (RR=0.95) and harm (RR=1.05).

f. Not able to assess inconsistency for CHD incidence as there was only one study available for inclusion.

g. Serious indirectness for CHD incidence as only one cohort available (EPIC-Oxford from the UK). In addition, there were no studies exclusive to or that included subgroup analyses in the diabetes population.

**2 References**

Appleby, P.N., Key, T.J., Thorogood, M., Burr, M.L., and Mann, J. (2002). Mortality in British vegetarians. *Public Health Nutr* 5(1)**,** 29-36. doi: 10.1079/PHN2001248.

Chang-Claude, J., Hermann, S., Eilber, U., and Steindorf, K. (2005). Lifestyle determinants and mortality in German vegetarians and health-conscious persons: results of a 21-year follow-up. *Cancer Epidemiol Biomarkers Prev* 14(4)**,** 963-968. doi: 10.1158/1055-9965.EPI-04-0696.

Crowe, F.L., Appleby, P.N., Travis, R.C., and Key, T.J. (2013). Risk of hospitalization or death from ischemic heart disease among British vegetarians and nonvegetarians: results from the EPIC-Oxford cohort study. *Am J Clin Nutr* 97(3)**,** 597-603. doi: 10.3945/ajcn.112.044073.

Guyatt, G.H., Oxman, A.D., Kunz, R., Woodcock, J., Brozek, J., Helfand, M., et al. (2011e). GRADE guidelines: 7. Rating the quality of evidence--inconsistency. *J Clin Epidemiol* 64(12)**,** 1294-1302. doi: 10.1016/j.jclinepi.2011.03.017.

Key, T.J., Appleby, P.N., Spencer, E.A., Travis, R.C., Roddam, A.W., and Allen, N.E. (2009). Mortality in British vegetarians: results from the European Prospective Investigation into Cancer and Nutrition (EPIC-Oxford). *Am J Clin Nutr* 89(5)**,** 1613S-1619S. doi: 10.3945/ajcn.2009.26736L.

Key, T.J., Fraser, G.E., Thorogood, M., Appleby, P.N., Beral, V., Reeves, G., et al. (1998). Mortality in vegetarians and non-vegetarians: a collaborative analysis of 8300 deaths among 76,000 men and women in five prospective studies. *Public Health Nutr* 1(1)**,** 33-41. doi:10.1079/PHN19980006.

Orlich, M.J., Singh, P.N., Sabate, J., Jaceldo-Siegl, K., Fan, J., Knutsen, S., et al. (2013). Vegetarian dietary patterns and mortality in Adventist Health Study 2. *JAMA Intern Med* 173(13)**,** 1230-1238. doi: 10.1001/jamainternmed.2013.6473.
